# Short-term Effect of Ambient Ozone on Daily Emergency Room Visits in Beijing, China

**DOI:** 10.1038/s41598-018-21154-x

**Published:** 2018-02-09

**Authors:** Yaohua Tian, Xiao Xiang, Juan Juan, Jing Song, Yaying Cao, Chao Huang, Man Li, Yonghua Hu

**Affiliations:** Department of Epidemiology and Biostatistics, School of Public Health, Peking University, No. 38 Xueyuan Road, 100191 Beijing, China

## Abstract

Little is known about the acute effects of ozone on morbidity risk in China. We conducted a time-series study to examine the association between ozone and daily emergency room visits (ERVs) in Beijing, China. We identified 7,088,309 ERVs between January 1, 2014 and December 31, 2015. A generalized additive model with Poisson regression incorporating penalized spline functions was employed to analyze ERVs in association with daily 8-h maximum ozone concentrations. An increase of 10 μg/m^3^ of same-day ozone concentration was significantly associated with a 0.24% (95% CI, 0.21%–0.26%), 0.31% (95% CI, 0.27%–0.35%), and 0.43% (95% CI, 0.36%–0.50%) increase in daily ERVs for the whole study period, days when the daily 8-h maximum ozone met the Chinese Ambient Air Quality Standards (CAAQS) Grade II standard, and days that met the CAAQS Grade I standard, respectively. These results were robust when considering the potential confounding effects of PM_2.5_, PM_10_, NO_2_, SO_2_, and CO. In conclusion, our findings suggested significant effects of ozone exposure on daily ERVs in Beijing. Improving air quality with even lower ozone level than the current CAAQS could yield important public health.

## Introduction

Short-term exposure to tropospheric ozone has been linked to increased mortality and morbidity^1–4^, accounting for an estimated 254,000 deaths globally in 2015^5^. The primary of previous studies evaluating the ozone-related health effects were conducted in Europe and North America^6–12^. Few studies have assessed the health effects of ozone exposure in developing countries. Given the considerable differences in ozone levels, weather patterns, topography, and population susceptibility across different geographic regions, an urgent need remains to assess the health effects of ambient ozone in developing countries.

China, the largest developing country, may have the severest ambient ozone pollution in the world^13^. Several studies have addressed the association between ozone and mortality in China^14–17^. However, little is known about impacts of ozone on morbidity outcomes in China. Emergency room visits (ERVs), an important measure of morbidity, greatly outnumber mortality events, thus having a greater statistical power to detect the air pollution-related health effects. In addition, ERV is almost impervious to external factors, such as availability of hospital beds and scheduled appointments with physicians. ERV can better test the temporal association between clinical presentation of disease and exposure to air pollution^18^. Therefore, ERV is a good indicator in evaluating the health effects associated with air pollution. Characterizing the association between exposure to ozone and ERV may help better delineate the scale and breadth of ozone’s impacts.

In 2012, the China’s Ministry of Environmental Protection proposed revisions to the Chinese Ambient Air Quality Standards (CAAQS) for ozone, adding a standard for the daily 8-h maximum ozone concentration. A key scientific issue in evaluating the ozone-associated health effects is whether a threshold concentration exists below which no adverse impacts are expected^1^. However, no study has specifically evaluated the health effects of ozone on morbidity risk at levels below the current CAAQS.

In this study, we aimed to test the relationship between short-term exposure to ozone and daily ERVs, and to explore whether ozone at levels below the current CAAQS has adverse effects on morbidity risk in Beijing, the capital of China.

## Methods

### Study population

Data on daily ERVs from January 1, 2014 to December 31, 2015 in Beijing were collected from Beijing Medical Claim Data for Employees, which covers urban employees who have basic medical insurance in Beijing. For each ERV, we extracted data on age, sex, and date of ERV. In 2016, the database covers a population of 18 million, accounting for >80% of the population in Beijing. This health database has been validated to be useful in environmental epidemiological studies^19,20^. The study was carried out in accordance with the Declaration of Helsinki. Because the data used for this study was collected for administrative purpose without any individual identifiers, this study was exempted from Institutional Review Board approval by the Ethics Committee of Peking University Health Science Center, Beijing, China. The need for informed consent was also waived by the Institutional Review Board.

### Air Pollution and Meteorological Data

Daily air pollution data, including ozone, particulate matter less than 2.5 μm in aerodynamic diameter (PM_2.5_), particulate matter less than 10 μm in aerodynamic diameter (PM_10_), nitrogen dioxide (NO_2_), sulfur dioxide (SO_2_), and carbon monoxide (CO) were obtained from a web platform (http://zx.bjmemc.com.cn/), which is run by the Beijing Environmental Protection Bureau. There were 35 monitors located in the 16 districts of Beijing. The daily average values for PM_2.5_, PM_10_, NO_2_, SO_2_, CO, and daily 8-h maximum ozone concentrations were calculated from the hourly observations across available monitoring stations. We also obtained meteorological data on daily mean temperature and relative humidity in Beijing from the China Meteorological Data Sharing Service System (http://data.cma.cn/).

### Statistical Analysis

Following a method used in a previous study^19^, a generalized additive Poisson model with penalized splines was fitted to analyze the association between ozone and ERVs. Several covariates were included in the model: (1) a degree of freedom (*df*) of 7 per year for calendar day to filter out seasonality^21^; (2) a 3 *df* for 3-day moving average relative humidity and temperature to accommodate non-linear and lag effects of meteorology^22^; (3) indicators for public holiday and day of week to adjust for the difference in the baseline ERVs for each day. Finally, we introduced the daily 8-h maximum ozone concentrations in the model.

To analyze the temporal association of ozone with ERVs, we fitted separate models with single–day lags (from lag 0 to lag 4) and multiday lag (lag 0–4). Smoothing function with 3 *df* was applied to analyze the exposure-response relationship between the log-relative risk of ERVs and ozone levels. We also explored the modifying effects of sex, age (18–64 and ≥ 65 years), and season (warm: April–September; and cool season: October–March). A Z-test was employed to assess the statistical significance of differences between subgroups^23^. Sensitivity analyses were conducted to assess the effect of *df* on the regression results. We also applied two-pollutant models to estimate the robustness of the association.

To explore whether there is evidence of a causal effect of ozone on morbidity even among individuals with ozone exposure at levels below the current CAAQS, we performed a subset approach that only includes days that meet the standards (Grade I standard of 100 μg/m^3^ and Grade II standard of 160 μg/m^3^).

All analyses were conducted in R 3.2.2 using the “*mgcv*” and “*nlme*” packages (R Foundation for Statistical Computing, Vienna, Austria). The results are expressed as percentage change and 95% confidence interval (CI) in daily ERVs per 10 μg/m^3^ increase of daily 8-h maximum ozone concentration.

## Results

Table 1 shows the basic characteristics for this study. A total of 7,088,309 ERVs between 2014 and 2015 in Beijing formed the basis for this study. There were 44.1% male patients, and 20.9% patients were ≥65 years old. Table 2 presents the summary statistics for daily ERVs and exposure variables during the study period. The mean (SD) daily ERV count was 9,710 (1070). The overall mean daily 8-h maximum ozone concentration was 103.1 μg/m^3^ with a range from 2.0 μg/m^3^ to 281.0 μg/m^3^. The means (SD) daily temperature and relative humidity were 15.9 °C (10.4 °C) and 55.0% (20.0%), respectively. Daily 8-h maximum ozone levels were weakly correlated with NO_2_, SO_2_, and CO concentrations (correlation coefficient r = −0.34–−0.29), while were highly positively correlated with temperature (r = 0.84) (Table 3).

**Table 1:** Demographic characteristics of emergency room visits in Beijing, 2014–2015.

Variable	No.
Total	7,088,309
Sex	
Male (%)	3,126,302 (44.1)
Female (%)	3,962,007 (55.9)
Age (year)	
18–64 (%)	5,605,708 (79.1)
≥65 (%)	1,482,601 (20.9)

**Table 2:** Summary statistics for daily counts of emergency room visits and exposure variables in Beijing, 2014–2015.

Variable	Mean ± SD	Minimum	Percentile			Maximum
			25th	50th	75th	
Emergency room visits	9710 ± 1070	7,591	9,166	9,731	10,388	15,527
Ozone (μg/m^3^)	103.1 ± 65.3	2	52	91	150	281
PM_2.5_ (μg/m^3^)	74.6 ± 61.7	5.2	28.9	56.6	104.3	328.3
PM_10_ (μg/m^3^)	97.9 ± 67.8	8.9	46.2	83.6	131.4	375.5
SO_2_ (μg/m^3^)	11.7 ± 13.3	2.0	3.4	6.9	14.1	79.0
NO_2_ (μg/m^3^)	48.2 ± 22.5	10.3	33.0	43.1	57.5	135.9
CO (mg/m^3^)	1.15 ± 0.84	0.22	0.62	0.94	1.34	6.82
Temperature (°C)	15.9 ± 10.4	−5.9	6.0	18.5	25.3	32.6
Relative humidity (%)	55 ± 20	8	41	56	70	99

PM_2.5_ = particulate matter with aerodynamic diameter <2.5 μm; PM_10_ = particulate matter with aerodynamic diameter <10 μm; SO_2_ = sulfur dioxide; NO_2_ = nitrogen dioxide; CO = carbon monoxide.

**Table 3:** Spearman correlation coefficients among the exposure variables.

Variables	ozone	PM_2.5_	PM_10_	NO_2_	SO_2_	CO	Temp	RH
ozone	1.00	−0.03	0.03	−0.34^a^	−0.30^a^	−0.29^a^	0.84^a^	0.04
PM_2.5_	—	1.00	0.83^a^	0.66^a^	0.53^a^	0.85^a^	−0.06	0.46^a^
PM_10_	—	—	1.00	0.68^a^	0.62^a^	0.67^a^	−0.04	0.13^b^
NO_2_	—	—	—	1.00	0.66^a^	0.74^a^	−0.32^a^	−0.16^a^
SO_2_	—	—	—	—	1.00	0.64^a^	−0.49^a^	−0.25^a^
CO	—	—	—	—	—	1.00	−0.29^a^	0.41^a^
Temp	—	—	—	—	—	—	1.00	0.23^a^
RH	—	—	—	—	—	—	—	1.00

PM_2.5_ = particulate matter with aerodynamic diameter <2.5 μm; PM_10_ = particulate matter with aerodynamic diameter <10 μm; SO_2_ = sulfur dioxide; NO_2_ = nitrogen dioxide; CO = carbon monoxide; Temp = temperature; RH = relative humidity.

^a^*P* < 0.001,

^b^*P* < 0.05.

**Figure 1:**
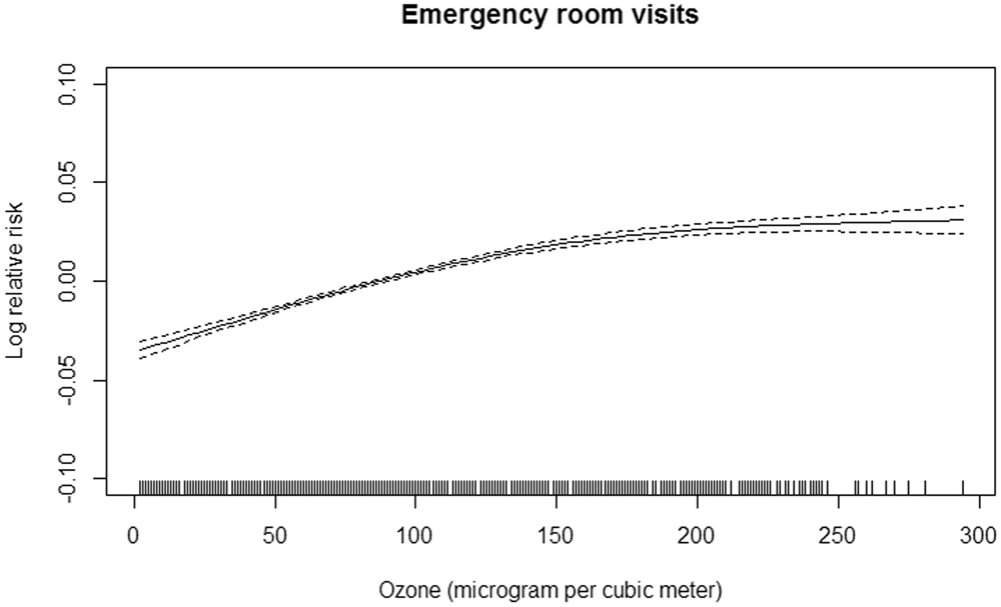
The exposure-response curve of same-day ozone concentrations and emergency room visits in Beijing, China. Note: The X-axis is the same-day 8-h maximum ozone concentrations (μg/m^3^). Y-axis is the predicted log (relative risk (RR)), after adjusting for temperature, relative humidity, day of week, public holiday, and calendar time, is shown by the solid line, and the dotted lines represent the 95% confidence interval.

As shown in Fig. 1, the exposure-response curve for daily 8-h maximum ozone concentrations and ERV was slightly non-linear with a sharp response at < 150 μg/m^3^, and a tiny fluctuation at higher levels. Table 4 shows the association between ERVs and ozone for different lag structures. We observed significant effects of zone on daily ERVs for the whole study period, days when the daily 8-h maximum ozone met the CAAQS Grade II standard, and days that met the CAAQS Grade I standard. A 10 μg/m^3^ increase in ozone concentration on the same day corresponded to a 0.24% (95% CI, 0.21%–0.26%), 0.31% (95% CI, 0.27%–0.35%), and 0.43% (95% CI, 0.36%–0.50%) increase in ERVs for the three study periods, respectively.

**Table 4:** Percentage changes with 95% confidence intervals (95% CIs) in emergency room visits associated with a 10 μg/m^3^ increase in ozone concentrations for different lag structures.

Hospital service	Lag days	Percentage change	95% CI	*P*
The whole study period	Lag 0 day	0.24	0.21–0.26	<2e-16
	Lag 1 day	0.09	0.06–0.11	4.23e-13
	Lag 2 day	0.06	0.04–0.09	4.59e-09
	Lag 3 day	0.05	0.03–0.08	3.78e-07
	Lag 4 day	0.01	−0.02–0.03	0.575
	Lag 0–4 day	0.23	0.19–0.26	<2e-16
Days that meet Grade II standard	Lag 0 day	0.31	0.27–0.35	<2e-16
(160 μg/m^3^)	Lag 1 day	0.06	0.03–0.09	0.000106
	Lag 2 day	0.07	0.04–0.09	7.57e-07
	Lag 3 day	0.08	0.05–0.11	3.46e-09
	Lag 4 day	0.02	−0.01–0.05	0.442
	Lag 0–4 day	0.27	0.22–0.31	<2e-16
Days that meet Grade I standard	Lag 0 day	0.43	0.36–0.50	<2e-16
(100 μg/m^3^)	Lag 1 day	0.16	0.11–0.20	2.7e-10
	Lag 2 day	−0.01	−0.05–0.03	0.583
	Lag 3 day	0.02	−0.02–0.06	0.298
	Lag 4 day	0.02	−0.02–0.06	0.322
	Lag 0–4 day	0.21	0.14–0.29	5.2e-08

The estimated effects varied with season and age groups (Table 5). A stronger association between ozone and ERVs was found in the warm season. The observed effects were larger for younger population. Altering the *dfs* for time and meteorological smoothers did not alter the estimated effects (Table 6). The estimated ozone effect on daily ERVs was robust when controlling for PM_2.5_, PM_10_. NO_2_, SO_2_, and CO (Table 7).

**Table 5:** Percentage changes with 95% confidence intervals (95% CIs) in emergency room visits associated with a 10 μg/m^3^ increase in same-day ozone concentration by sex, age, and season.

	Percentage change	95% CI	*P*-value
Sex			0.166
Male	0.25	0.22–0.29	
Female	0.22	0.19–0.26	
Age (year)			0.044
18–64	0.25	0.22–0.27	
≥65	0.19	0.14–0.24	
Season^a^			<0.001
Warm	0.33	0.30–0.36	
Cool	0.06	0.01–0.12	

^a^Warm season: from April to September; cool season: from October to March.

**Table 6:** Percentage changes with 95% confidence intervals (95% CIs) in emergency room visits associated with a 10 μg/m^3^ increase in same-day ozone concentration, by different degree of freedom (*df*) per year for calendar time, temperature, and relative humidity.

Variable	*df*	Percentage change	95% CI	*P* value
Emergency room visits				
Calendar time	5	0.27	0.24–0.29	<2e-16
	6	0.27	0.24–0.29	<2e-16
	7^a^	0.24	0.21–0.26	<2e-16
	8	0.17	0.14–0.19	<2e-16
	9	0.17	0.15–0.20	<2e-16
Temperature	2	0.24	0.21–0.26	<2e-16
	3^a^	0.24	0.21–0.26	<2e-16
	4	0.23	0.21–0.26	<2e-16
	5	0.22	0.19–0.24	<2e-16
	6	0.22	0.20–0.25	<2e-16
Relative humidity	2	0.24	0.21–0.26	<2e-16
	3^a^	0.24	0.21–0.26	<2e-16
	4	0.23	0.21–0.26	<2e-16
	5	0.23	0.20–0.25	<2e-16
	6	0.23	0.21–0.25	<2e-16

^a^The *df* value used in this study model.

**Table 7:** Percentage change with 95% CI in emergency room visits associated with a 10 μg/m^3^ increase in same-day ozone concentration in two-pollutant models.

Variable	Percentage change	95% CI	*P*-value
Adjust PM_2.5_	0.23	0.20–0.25	<2e-16
Adjust PM_10_	0.23	0.20–0.25	<2e-16
Adjust SO_2_	0.27	0.25–0.30	<2e-16
Adjust NO_2_	0.24	0.22–0.27	<2e-16
Adjust CO	0.25	0.22–0.27	<2e-16

## Discussion

Evidence gained in this analysis indicated that the current level of ozone in Beijing was significantly associated with increased daily ERVs, even at ozone levels below the current CAAQS. The association was robust when considering the potential confounding effects of PM_2.5_, PM_10_, NO_2_, SO_2_, and CO. The ozone-related health effects are of increasing concern to the public in China^13,24^. Our findings should contribute to the limited scientific data and may have some implications for policy making and standards setting in China.

The effect of ambient ozone on mortality risk has been extensively reported in previous studies^2,25,26^. However, the association between ozone and morbidity risk is less studied and the findings are inconsistent. In Canada, a study involving 720,519 hospitalizations for respiratory disease in 16 cities reported significant effects of previous day’s 1-hour maximum ozone concentration on morbidity risk in the April to December period^27^. In contrast, a study in Windsor, Ontario, Canada, failed to find a significant association of ozone with respiratory admissions^28^. A time-series study in 8 French cities suggested that short-term exposure to ozone was not associated with daily hospitalizations for cardiovascular diseases^8^. Similarly, Barnett *et al*.^29^ demonstrated that ozone showed no significant association with cardiovascular disease admissions in subjects aged 65 years and over in 7 Australian and New Zealand cities. Son *et al*.^30^ reported increased daily hospital admissions for allergic disease, asthma and selected respiratory disease in relation to ozone exposures in 8 Korean cities. A meta-analysis demonstrated significant associations of ambient ozone with various types of respiratory hospitalizations, although heterogeneity across studies’ results was observed^4^. The heterogeneity of findings may reflect differences in the characteristics of ozone pollution, outcome definitions, weather conditions, and population susceptibility.

Overall, we found a significant association between ozone and ERVs, which is consistent with previous studies on the effects of ozone on mortality risk in China^14–17,31^. A meta-analysis of five studies on the association between ozone and mortality in China demonstrated that a 10 μg/m^3^ increase in daily 8-h maximum ozone concentrations was associated with a 0.42% (95% CI, 0.32%–0.52%), 0.44% (95% CI, 0.17%–0.70%), and 0.50% (95% CI, 0.22%–0.77%) increase in daily non-accidental, cardiovascular, and respiratory mortality, respectively^26^. However, few studies have assessed the acute effects of ozone on morbidity risk in China, perhaps due to the lack of ozone monitoring and morbidity data.

In this study, we found that the ambient ozone had stronger effects on ERVs in the warm season than in the cool season. This finding is consistent with most previous studies in western countrie^4,32^, but contradicts several studies in China that reported stronger mortality effects of ozone in cool season^14,16,17^. In this analysis, the correlation analysis showed that ozone levels were highly positively correlated with temperature. The overall mean daily 8-h maximum ozone concentration during warm season was much higher than that during cool season (147.8 μg/m^3^ vs. 50.2 μg/m^3^). It is plausible that the greater ozone effects in the warm season may attributable to the higher ozone levels during warm season. This hypothesis is supported by a study conducted in the Pearl River Delta of southern China demonstrating higher estimated effects of ozone on total and cardiovascular mortality during peak exposure periods (September through November)^15^. In addition, differences in window opening frequencies and outdoor activity patterns during warm and cool season may also be responsible for the seasonal pattern of ozone effects.

The subset analysis showed that the estimated ozone effects were stronger in days with lower ozone levels, which is complemented by the sharp slop at lower ozone levels shown in the exposure-response curve. Generally, our findings were consistent with previous studies on the health effects of air pollution^21,33^. For example, a recent nationwide time-series study in 272 Chinese cities demonstrated that the association between PM_2.5_ and mortality risk were stronger in cities with lower PM_2.5_ concentrations^21^. The relatively weaker effects at higher ozone levels might be related to “harvesting effect” in that people who are susceptible to ozone exposure might have developed symptoms and went to hospitals before ozone concentration reached a fairly high level^34^.

In 2012, a standard for the daily 8-h maximum ozone concentration was established in China. However, no study to date has characterized the health effects of ozone on morbidity risk at levels below the regulatory standard. In this study, we found that short-term exposure to ozone could still significantly increases daily ERVs for days that met the current CAAQS. A recent study conducted in Jiangsu Province, China, indicated that short-term exposure to ozone could increase mortality risk, even for ozone levels not exceeding the current CAAQS Grade II standard^16^. These evidences indicate that reduction in ozone levels in Beijing, even in cities that meet the current air quality standard, would generate substantial health benefits.

A critical issue concerning the association between ozone and daily ERVs is the extent to which this association was confounded by other air pollutants. In this analysis, daily ozone concentrations were weakly correlated with PM_2.5_, PM_10_, NO_2_, SO_2_, and CO (correlation coefficient r = −0.34 –0.03), thus reducing their likelihood as confounders. A prior study has demonstrated that the correlation between ozone and other air pollutants is often low, thus the effects of ozone and other air pollutants can be separately relatively easily^1^. In addition, we fitted two-pollutant models to estimate the robustness of the association between ozone and daily ERVs. The effect estimates changed little after adjustment of other air pollutants. The robustness of the effect estimates with inclusion of co-pollutants suggested independent effects of ozone on daily ERVs, which is consistent with previous findings^35,36^.

The estimated ozone effects were relatively lower in the elderly in this analysis, which contradicts the primary of previous studies. Most studies reported stronger effects of ozone in the elderly^25^, while two nationwide studies in the U.S. and China did not find evidence for effect modification by age^6,37^. Moreover, a study conducted in Shanghai, China examined the association between short-term exposure to ozone and daily mortality, and found that the effect estimate was lower in people aged ≥65 years than in people aged 45–64 years^14^. In China, the elderly are advised to minimize or avoid outdoor activity or wear a face mask outdoors when air pollution is severe, thus decreasing personal exposure^38^. In contrast, younger populations are expected to spend more time outdoors, thus monitoring measurements could more accurately reflect personal exposure. This may cover up the real age-specific effect of ozone. In addition, the results for age class could be driven by the inclusion of all-cause emergency room visits. The patterns for age-specific ozone effects may varied by specific diseases. A meta-analysis explored the modifiers of ozone effects, and found that estimates for cardiovascular diseases were higher for older group than for younger group, but the estimates for respiratory mortality and asthma admission were lower for older groups^25^. Future studies are warranted to explore the age-specific effects of ozone on ERVs for specific diseases. The ozone exposure showed a marked temporal association with ERVs. The strongest effects were observed at lag 0, with this association diminishing at longer lag times. Although the magnitude of risk estimates was relatively small, the health burden attributable to ambient ozone pollution in China may be heavy because the overwhelming majority of the public is exposed to ozone, indicating potentially large public health implications.

There are several limitations in the present study. First, the use of citywide average ozone levels to represent individual exposure is expected to cause exposure measurement error. A study has demonstrated that this exposure measurement error would most likely to bias the risk estimates downward^39^. Second, we were not able to analyze the association between ozone and cause-specific disease because data on clinical diagnosis were not available in our database. Another limitation is our inability to adjust for the confounding role of flu epidemics in the association between short-term exposure to ozone and health. Finally, the only inclusion of active urban employers and retired subjects with basic medical insurance might introduce a selection bias. This bias could lead to an underestimation of the real effect.

In conclusion, we estimated significant increase in daily ERVs associated with ozone exposure in Beijing, even for exposure levels below the current CAAQS. Our findings strengthen the rationale for further limiting ambient ozone levels in Beijing.
